# Evaluation of periodontitis-related tooth loss according to the new 2018 classification of periodontitis

**DOI:** 10.1038/s41598-022-15462-6

**Published:** 2022-07-13

**Authors:** Masahide Takedachi, Yoshio Shimabukuro, Keigo Sawada, Mami Koshimizu, Kazuko Shinada, Harumi Asai, Ayumi Mizoguchi, Yuko Hayashi, Akina Tsukamoto, Midori Miyago, Fuuka Nishihara, Takako Nishihata, Michiyo Shimabukuro, Hiroyuki Kurakami, Tomoharu Sato, Yayoi Hamazaki, Tomoaki Iwayama, Chiharu Fujihara, Shinya Murakami

**Affiliations:** 1 Department of Periodontology, Osaka University Graduate School of Dentistry, Osaka, Japan; 2 Shimabukuro Dental Clinic, 1-5-6 Tezukayamahigashi, Sumiyoshi-ku, Osaka, 558-0054 Japan; 3 Department of Medical Innovation, Osaka University Hospital, Osaka, Japan; 4 Department of Biostatistics and Data Science, Osaka University Graduate School of Medicine, Osaka, Japan

**Keywords:** Prognostic markers, Dental diseases

## Abstract

The new 2018 classification of periodontal diseases is reported to be related to tooth loss due to periodontal disease (TLPD) during supportive periodontal therapy (SPT). However, few reports have evaluated this relationship for Asians or have analyzed the association of the new classification and TLPD by distinguishing between active periodontal therapy (APT) and SPT. In this study, we retrospectively applied the new classification to 607 Japanese periodontitis patients and examined the relationship between the new classification and annual TLPD rates per patient during the respective periods. TLPD rates were higher in patients in stage IV and/or grade C during both APT and SPT. TLPD during SPT was not associated with the presence or absence of TLPD during APT. Multivariate analysis revealed that stage IV and grade C as independent variables were significantly associated with the number of instances of TLPD not only during the total treatment period, but also during APT or SPT. Our results suggest that the new classification has a significantly strong association with TLPD during both APT and SPT, and that patients diagnosed with stage IV and/or grade C periodontitis had a higher risk of TLPD during both periods.

## Introduction

Periodontitis is an inflammatory disease caused by bacterial biofilms on the dental root surface, and is one of main causes of tooth loss due to irreversible destruction of the tooth-supporting tissues (periodontal tissues). However, active periodontal therapy (APT), including non-surgical debridement and surgical periodontal therapy, and continued maintenance in the form of supportive periodontal therapy (SPT), based on an accurate diagnosis, enable teeth to be preserved for a long time^1–6^. Hence, the development of diagnostic or predictive models that can determine the prognosis and guide periodontal treatment would be useful for periodontists^3,7,8^.

Periodontitis was classified into chronic periodontitis and aggressive periodontitis in 1999 by the American Academy of Periodontology (AAP)^9^, and this classification has been widely used for many years. To overcome some of the limitations of this classification, a world workshop co-organized by the AAP and the European Federation of Periodontology was held in 2017 and a new 2018 classification of periodontitis characterized by a multidimensional staging and grading system^10^ was developed. In the new 2018 classification, periodontitis was divided into four stages according to severity and disease management complexity (stages I–IV; stage I is the mildest, stage IV is the most severe), while the risk of disease progression was denoted by three grades (grades A–C; grade A is the lowest risk, grade C is the highest risk) based on radiographic bone loss, clinical attachment loss, and risk factors such as smoking and diabetic status. In addition, the extent of each stage was classified as localized or generalized. Since the new 2018 classification was promulgated, it has been widely introduced in research, education, and clinical practice^11–18^. With the popularization of the new 2018 classification, a relationship between diagnosis based on the new classification and prognosis based on tooth loss due to periodontal disease (TLPD) has been reported in cohort studies on Westerners^15–18^. Although these studies showed that the new 2018 classification can be used as a risk assessment for periodontitis, there is currently insufficient information to generalize the use of this classification for the prognosis of periodontitis.

Periodontal therapy is performed on the basis of the initial diagnosis and treatment protocol. Teeth that are not expected to respond to APT and are considered to have a poor prognosis are extracted. Additionally, some teeth are extracted due to prosthetic requirements for functional restorative treatment. Once the periodontal tissue is stabilized during APT, SPT is initiated to preserve the teeth over the long term. The prognosis after the completion of periodontal therapy is evaluated by TLPD during SPT, and is considered to be related to the APT protocol^5,19,20^. However, although there are sufficient reports on factors that may affect the prognosis, including the initial diagnosis and prognosis estimated during SPT, few reports are available that determine the prognosis by taking TLPD during APT into account^5,19,20^.

In this study, we investigated whether the new 2018 classification at the start of APT can be used to judge the prognosis of periodontal disease using TLPD when targeting Japanese patients. In addition, by conducting the analysis separately for APT and SPT, we aimed to verify the importance of the new 2018 classification as a prognostic index in each treatment period. To address these questions, we retrospectively applied the new classification to patients attending a private dental clinic in Japan, and then examined the distribution of stages and grades among the patients, as well as baseline characteristics, to investigate annual TLPD during the respective periods.

## Results

### Patient characteristics

A total of 607 patients were included in this study, with a mean age of 54.4 ± 11.9 years and a mean number of teeth present of 26.1 ± 3.7 at baseline. The total duration (months) of the whole treatment period, APT period, and SPT period was 80.9 ± 34.2 (range: 16 to 190 months), 11.1 ± 6.4 (range: 2 to 35 months), and 69.9 ± 35.3 (range: 12 to 174 months), respectively (Table 1). There was no statistically significant difference between men and women regarding tooth number and treatment periods in this study (Table 1). Twelve participants (2.0%) had diabetes, 43 participants (7.1%) were active smokers, and 93 (15.3%) were former smokers. There was no statistically significant difference in the total treatment period (APT + SPT) and the respective periods of APT and SPT due to differences in sex, diabetes, or smoking status in this study (data not shown).

**Table 1:** Patient characteristics and treatment duration in total and by sex.

	Male (n = 179)		Female (n = 428)		*p-*value	Total (n = 607)	
	Mean ± SD	Median (25% quantile, 75%quantile)	Mean ± SD	Median (25% quantile, 75%quantile)		Mean ± SD	Median (25% quantile, 75%quantile)
Age (year)	55.3 ± 12.0	57.0 (48.0, 63.0)	54.0 ± 11.8	53.0 (46.0, 62.0)	0.18*	54.4 ± 11.9	54.0 (47.0, 62.5)
Number of teeth	26.3 ± 3.7	25.0 (25.0, 28.0)	26.0 ± 3.7	27.0 (24.0, 28.0)	0.37^†^	26.1 ± 3.7	27.0 (25.0, 28.0)
APT + SPT duration (months)	81.7 ± 32.9	81.0 (54.0, 108.0)	80.7 ± 34.7	77.5 (52.0, 107.3)	0.59^†^	80.9 ± 34.2	80.0 (53.0, 108.0)
APT duration (months)	11.2 ± 6.7	10.0 (7.0, 14.0)	11.0 ± 6.3	10.0 (7.0, 14.0)	0.96^†^	11.1 ± 6.4	10.0 (7.0, 14.0)
SPT duration (months)	70.5 ± 35.4	66.0 (42.0, 96.5)	69.6 ± 36.1	68.0 (40.5, 96.3)	0.73^†^	69.9 ± 35.3	67.0 (41.0, 96.5)

Statistical analysis between the two groups with age was performed with Student’s *t* test. Statistical analysis between the two groups with other variables was performed with the Mann–Whitney U test.

*SD* standard deviation; *APT* active periodontal therapy; *SPT* supportive periodontal therapy.

*p-value from Student’s *t* test.

^†^*p-*value from the Mann–Whitney U test.

### Categorization of patients according to the new 2018 classification

**Figure 1:**
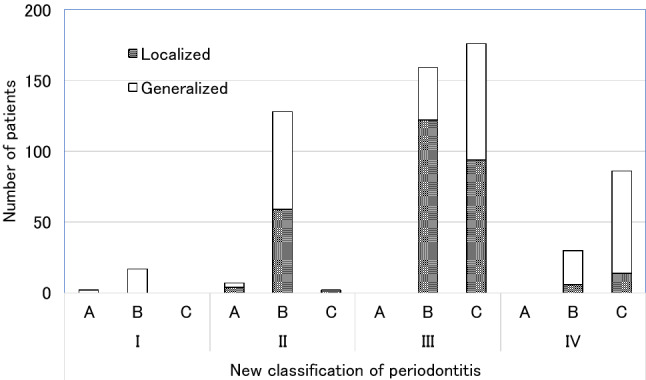
Distribution of periodontitis patients according to the new 2018 classification of periodontitis.

We retrospectively applied the new 2018 classification to all the patients. One hundred seventy-six patients (29.0%) were classified into stage III grade C, followed by 159 (26.2%) in stage III grade B, and 128 (21.1%) in stage II grade B. A higher proportion of the patients in stage III and stage IV were classified into grade C, and most of those in stage II were classified into grade B. A higher proportion of the patients in stage III had localized periodontitis, whereas those in stage IV were more likely to have generalized periodontitis. The proportion of localized and generalized periodontitis in the stage II patients was almost equal (Fig. 1). The number of teeth at the start of APT of the patients who were classified into the respective stages, grades, and extents are shown in Table 2. The Kruskal–Wallis test detected significant differences in the number of teeth between stages and grades. The number of teeth present at the initial diagnosis was analyzed with the Steel–Dwass post hoc test or the Mann–Whitney U test. The results indicated that the number of teeth was significantly lower in patients classified at a higher stage. It was also smaller in those classified at a higher grade; however, a significant difference was only noted between those with grade B and grade C. Interestingly, compared with the patients with stage IV disease, the number of teeth present was significantly greater in grade C patients than in grade B patients. Patients with generalized periodontitis had a significantly lower number of teeth present than those with localized periodontitis.

**Table 2:** Number of teeth at baseline by stage, grade, and extent.

Stage	Number of patients (%)	Number of teeth at T0	*p-*value	Grade	Number of patients (%)	Number of teeth at T0	*p-*value	Extent	Number of patients (%)	Number of teeth at T0	*p-*value	Stage	Grade A		Grade B		Grade C		*p*-value
													Number of patients	Number of teeth at T0	Number of patients	Number of teeth at T0	Number of patients	Number of teeth at T0	
I	19 (3.1)	28.8 ± 2.1	< 0.01^†^	A	9 (1.5)	28.0 ± 3.0	< 0.01^†^	Localized	300 (49.4)	27.1 ± 2.2	< 0.01^§^	I	2	28.0 ± 0.0	17	28.9 ± 2.2	0	NA	0.47^§^
II	137 (22.6)	27.5 ± 2.2		B	334 (55.0)	26.5 ± 3.6		Generalized	307 (50.6)	25.1 ± 4.5		II	7	28.0 ± 3.4	128	27.4 ± 2.1	2	29.0 ± 1.4	0.46^†^
III	335 (55.2)	26.9 ± 2.2^ab^		C	264 (43.5)	25.5 ± 3.8^e^						III	0	NA	159	26.9 ± 2.4	176	26.9 ± 2.1	0.70^§^
IV	116 (19.1)	21.6 ± 5.0^acd^										IV	0	NA	30	18.9 ± 5.3	86	22.6 ± 4.6	< 0.01^§^
Total	607 (100.0)	26.1 ± 3.7		Total	607 (100.0)	26.1 ± 3.7		Total	607 (100.0)	26.1 ± 3.7									

Statistical analysis comparing three or four groups according to the number of teeth was performed with the Kruskal–Wallis test followed by the Steel–Dwass post hoc test. Statistical analysis comparing two groups according to the number of teeth was performed with the Mann–Whitney U test.

T0, start of APT.

Number of teeth at T0 is shown as mean ± standard deviation.

^†^*p*-value from the Kruskal–Wallis test.

^§^*p*-value from the Mann–Whitney U test.

Significant difference by the Steel–Dwass post hoc test compared with Stage I (*p* < 0.01:a), Stage II (*p* < 0.05:b, *p* < 0.01:c), Stage III (*p* < 0.01:d), and Grade B (*p* < 0.01:e).

### Relationship between the new 2018 classification and TLPD

One hundred thirty patients (21.4%) out of a total of 607 patients lost teeth during the whole treatment period. During the total treatment period, 260 teeth (63 during APT and 197 during SPT) out of 15,838 that existed at the initial diagnosis were lost. Of the 42 patients (6.9%) who had TLPD during APT, 16 (2.6%) also lost teeth during SPT. Additionally, 88 (15.5%) out of the 565 patients who had not experienced TLPD during APT lost teeth during SPT. The incidence of tooth loss in each stage, grade, and extent is presented in Supplemental Table 1. The mean annual TLPD rate per patient was 0.07 ± 0.18 during the total treatment period. We then also examined the mean annual TLPD rates per patient by the respective stages and grades according to the new 2018 classification (Table 3 and Supplemental Table 2). The Kruskal–Wallis test detected significant differences between the groups. Patients in stage I, stage II, and grade A had no TLPD during the total treatment period. Patients in stage IV, grade C had TLPD rates of 0.24 ± 0.31, 0.15 ± 0.24 (number of teeth/patient/year), respectively, with significant differences when compared with those in the other stages and grades. During both APT and SPT, similar to TLPD rates for respective stages and grades during the total treatment period, the patients in stage IV had the highest TLPD in the stage classification, along with those in grade C in the grade classification. The annual tooth loss per patient during SPT was generally approximately half of the corresponding stage or grade during APT. To determine whether TLPD during APT was associated with TLPD during SPT, the differences among TLPD rates during SPT were compared on the basis of the presence or absence of TLPD during APT (Table 4). The results revealed no statistically significant difference in TLPD rates during SPT with the presence or absence of TLPD during APT in any patients who had TLPD in stage III, stage IV, grade B, or grade C in this study.

**Table 3:** Annual tooth loss per patient for each stage and grade during APT + SPT, APT, and SPT.

		APT + SPT			APT			SPT		
	Number of patients	Mean ± SD	Median (25% quantile; 75% quantile)	*p-*value	Mean ± SD	Median (25% quantile; 75% quantile)	*p-*value	Mean ± SD	Median (25% quantile; 75% quantile)	*p-*value
**Stage**										
I	19	0.00 ± 0.00	0.00 (0.00;0.00)	< 0.01*	0.00 ± 0.00	0.00 (0.00;0.00)	< 0.01*	0.00 ± 0.00	0.00 (0.00;0.00)	< 0.01*
II	137	0.00 ± 0.00	0.00 (0.00;0.00)		0.00 ± 0.00	0.00 (0.00;0.00)		0.00 ± 0.00	0.00 (0.00;0.00)	
III	335	0.04 ± 0.11^c^	0.00 (0.00;0.00)		0.07 ± 0.33^b^	0.00 (0.00;0.00)		0.05 ± 0.14^c^	0.00 (0.00;0.00)	
IV	116	0.24 ± 0.31^acd^	0.14 (0.00;0.34)		0.39 ± 0.85^ cd^	0.00 (0.00;0.00)		0.18 ± 0.27^acd^	0.00 (0.00;0.30)	
**Grade**										
A	9	0.00 ± 0.00	0.00 (0.00;0.00)	< 0.01*	0.00 ± 0.00	0.00 (0.00;0.00)	< 0.01*	0.00 ± 0.00	0.00 (0.00;0.00)	< 0.01*
B	334	0.01 ± 0.05	0.00 (0.00;0.00)		0.02 ± 0.17	0.00 (0.00;0.00)		0.01 ± 0.03	0.00 (0.00;0.00)	
C	264	0.15 ± 0.24^ef^	0.08 (0.00;0.28)		0.23 ± 0.66	0.00 (0.00;0.00)		0.13 ± 0.23^f^	0.00 (0.00;0.17)	
Total	607	0.07 ± 0.18	0.00 (0.00;0.00)		0.11 ± 0.46	0.00 (0.00;0.00)		0.06 ± 0.17	0.00 (0.00;0.00)	

Statistical analysis between the three or four groups was performed with the Kruskal–Wallis test followed by the Steel–Dwass post hoc test.

*SD* standard deviation, *APT* active periodontal therapy, *SPT* supportive periodontal therapy.

*p-value from the Kruskal–Wallis test.

Significant difference by the Steel–Dwass post hoc test compared with Stage I (*p* < 0.01:a), compared with Stage II (*p* < 0.05:b, *p* < 0.01:c), compared with Stage III (*p* < 0.01:d), compared with Grade A (*p* < 0.05: e), compared with Grade B (*p* < 0.01: f).

0.00 in the table shows that the number of extracted tooth was zero.

**Table 4:** Annual tooth loss per patient during SPT with or without tooth loss during APT.

		Patients who did not lose any teeth during APT			Patients who lost a tooth during APT			
			Annual tooth loss per patient during SPT			Annual tooth loss per patient during SPT		
	Number of patients	Number of patients	Mean ± SD	Median (25% quantile;75% quantile)	Number of patients	Mean ± SD	Median (25% quantile;75% quantile)	*p-*value
**Stage**								
I	19	19	0.00 ± 0.00	0.00 (0.00;0.00)	0			
II	137	137	0.00 ± 0.00	0.00 (0.00;0.00)	0			
III	335	319	0.04 ± 0.14	0.00 (0.00;0.00)	16	0.05 ± 0.08	0.00 (0.00;0.11)	0.30
IV	116	90	0.17 ± 0.25	0.00 (0.00;0.31)	26	0.18 ± 0.32	0.00 (0.00;0.26)	0.70
**Grade**								
A	9	9	0.00 ± 0.00	0.00 (0.00;0.00)	0			
B	334	329	0.00 ± 0.03	0.00 (0.00;0.00)	5	0.05 ± 0.11	0.00 (0.00;0.00)	0.46
C	264	227	0.13 ± 0.23	0.00 (0.00;0.16)	37	0.14 ± 0.28	0.00 (0.00;0.17)	0.11
Stage I Grade A	2	2	0.00 ± 0.00	0.00 (0.00;0.00)	0			
Stage I Grade B	17	17	0.00 ± 0.00	0.00 (0.00;0.00)	0			
Stage II Grade A	7	7	0.00 ± 0.00	0.00 (0.00;0.00)	0			
Stage II Grade B	128	128	0.00 ± 0.00	0.00 (0.00;0.00)	0			
Stage II Grade C	2	2	0.00 ± 0.00	0.00 (0.00;0.00)	0			
Stage III Grade B	159	155	0.00 ± 0.02	0.00 (0.00;0.00)	4	0.06 ± 0.12	0.00 (0.00;0.06)	0.06
Stage III Grade C	176	164	0.08 ± 0.19	0.00 (0.00;0.08)	12	0.05 ± 0.07	0.00 (0.00;0.10)	0.76
Stage IV Grade B	30	29	0.03 ± 0.07	0.00 (0.00;0.00)	1	0.00 ± 0.00	0.00 (0.00;0.00)	0.77
Stage IV Grade C	86	61	0.24 ± 0.27	0.00 (0.00;0.38)	25	0.19 ± 0.32	0.00 (0.00;0.26)	0.19
Total	607	565	0.05 ± 0.16	0.00 (0.00;0.00)	42	0.13 ± 0.26	0.00 (0.00;0.16)	

Statistical analysis was performed with the Mann–Whitney U test. Tooth loss per patient is shown as mean ± standard deviation. *APT* active periodontal therapy; *SPT* supportive periodontal therapy.

Tables 5, 6, and 7 summarize the outcomes of negative binomial regression analysis of the association between the new 2018 classification and TLPD during the total treatment period (APT + SPT), APT, and SPT, respectively. The independent variables included sex, age, number of teeth present at baseline, stages, grades, and extents. The analysis demonstrated that stage IV, grade C, and generalized extent were significantly associated with TLPD during the total treatment period (Table 5). Additionally, we performed the same analysis during the APT period only (Table 6) and during the SPT period only (Table 7). The results showed that stage IV and grade C, but not generalized extent, were significantly associated with TLPD during APT (Table 6). Furthermore, stage IV, grade C, and generalized extent were significantly associated with TLPD during SPT (Table 7). Although the number of instances of TLPD during APT was also used as an independent variable for the analysis of TLPD during SPT, the association was not statistically significant.

**Table 5:** Results of multivariate analysis (negative binomial regression) of number of extraced teeth during APT + SPT.

Independent variable	IRR (95%CI)	*p-*value
**Sex**		
Female	Reference	
Male	1.10 (0.27–1.46)	0.40
Age	1.51 (0.02–2.04)	0.57
Number of teeth at T0	0.69 (0.01–1.18)	0.19
**Stage**		
I, II, & III	Reference	
IV	1.38 (1.03–1.78	< 0.01
**Grade**		
A & B	Reference	
C	2.57 (2.05–3.16)	< 0.01
**Extent**		
Localized	Reference	
Generalized	1.26 (1.03–1.47)	< 0.01

Multivariate analysis (negative binomial regression) of the number of extracted teeth due to periodontitis per patient during APT + SPT; analysis was corrected for the period of follow-up.

*APT* active periodontal therapy; *SPT* supportive periodontal therapy; *IRR* incidence rate ratio; *CI* confidence interval; *T0* start of APT.

**Table 6:** Multivariate analysis (negative binomial regression) of number of extraced teeth during APT.

Independent variable	IRR (95%CI)	*p-*value
**Sex**		
Female	Reference	
Male	0.30 (0.02–1.18)	0.37
Age	1.52 (0.24–2.07)	0.19
Number of teeth at T0	0.62 (0.09–1.12)	0.28
**Stage**		
I, II, & III	Reference	
IV	1.92 (1.54–2.77)	< 0.01
**Grade**		
A & B	Reference	
C	2.01 (1.08–3.12)	< 0.01
**Extent**		
Localized	Reference	
Generalized	0.43 (0.05–1.26)	0.43

Multivariate analysis (negative binomial regression) of the number of extracted teeth due to periodontitis per patient during APT; analysis was corrected for the period of follow-up.

*APT* active periodontal therapy; *IRR* incidence rate ratio; *CI* confidence interval; *T0* start of APT.

**Table 7:** Multivariate analysis (negative binomial regression) of the number of extracted teeth during SPT.

Independent variable	IRR (95%CI)	*p-*value
**Sex**		
Female	Reference	
Male	0.68 (0.04–1.36)	0.36
Age	1.02 (0.00–2.04)	0.27
Number of teeth at T0	0.44 (0.02–1.10)	0.18
Number of teeth lost during APT	1.18 (0.55–3.15)	0.28
**Stage**		
I, II, & III	Reference	
IV	1.78 (1.23–2.69)	< 0.01
**Grade**		
A & B	Reference	
C	2.72 (2.10–3.42)	< 0.01
**Extent**		
Localized	Reference	
Generalized	1.43 (1.20–1.82)	< 0.01

Multivariate analysis (negative binomial regression) of the number of extracted teeth due to periodontitis per patient during SPT; analysis was corrected for the period of follow-up.

*APT* active periodontal therapy; *SPT* supportive periodontal therapy; *IRR* incidence rate ratio; *CI* confidence interval; *T0* start of APT.

## Discussion

The aim of this study was to clarify the relationship between the new 2018 classification of periodontitis and TLPD during APT and SPT in 607 patients who had received periodontal therapy for 16 to 190 months (mean 69.9 months) in a private dental clinic in Japan. Previous reports demonstrating the association of the new 2018 classification with TLPD only analyzed TLPD during SPT^16^. To the best of our knowledge, this is the first study that takes TLPD during ATP into account. Our results revealed that TLPD rates during the total treatment period were higher in higher stages and grades. Moreover, this relationship between TLPD and stage/grade was observed both during APT and SPT, indicating that the new 2018 classification has predictive ability for periodontal prognosis with TLPD as an index during the respective periods. Because an accurate prediction of the periodontal prognosis plays an important role over the long term from the time of development of a treatment protocol to SPT, the application of the new 2018 classification to the prognosis of periodontitis has been investigated in studies of Western patients attending university dental hospitals^15,16^. This study helps to generalize the use of the new 2018 classification for prognosis by targeting different types of subjects in Japan.

Limitations of this study include the fact that only patients who had received active SPT were covered, and that there was a lack of variation among patients. It is known that if periodontitis is left untreated, it progresses with age^21^, and irregular compliers under SPT, even after the completion of periodontal treatment, have a worse prognosis than regular compliers^6,22–24^. It is uncertain whether the relationship between the new 2018 classification and TLPD would be similar to the results of this study if proper SPT was not provided or if periodontal therapy was not received. Additionally, a relatively small number of subjects were smokers or patients with diabetes. Smoking and diabetes are associated with the rate of periodontitis progression according to the new 2018 classification. Patients who are health-conscious and receive SPT for a long period are also likely to have ceased smoking and be receiving diabetes treatment. Therefore, if a population with a large proportion of smokers and/or patients with diabetes is targeted, the grade determined at the initial diagnosis may be less closely associated with TLPD rates during SPT.

Because periodontitis spreads and progresses with TLPD with increasing age^25–27^, the mean age of the patients was higher and the number of teeth present was lower at the higher stages, and there was also a significant difference in age between those with localized and generalized periodontitis in this study (data not shown). Because the grade is defined by the bone resorption rate/age, and bone resorption rates are often at the same level at the same stage, younger patients are classified at a higher grade in the same stage. This results in the mean age significantly decreasing as the grade progresses in stage II, III, and IV. Furthermore, the progression of periodontitis with age in association with TLPD explains why the number of teeth in grade C patients was significantly higher than that in grade B patients at stage IV.

Martinez-Caunt et al.^28^ and Ravald et al.^29^ reported that the number of teeth present at the initiation of SPT was associated with overall tooth loss including causes other than periodontal disease. In regard to the rate of TLPD to overall tooth loss, the former report showed 72.5% and the latter report showed 58.9%; however, the relationship between the number of teeth present at baseline and TLPD during SPT was not investigated. In this study, we revealed that the number of teeth present at baseline was not associated with TLPD during SPT. In addition, multivariate analysis with the number of teeth at start of SPT as an independent variable in the same subjects showed no association with TLPD (data not shown). These results were consistent with other results obtained in this study which demonstrated that tooth loss during APT was not significantly associated with TLPD during SPT in either stages or grades. This indicates that TLPD during SPT depends not on the number of teeth present but on other factors related to the new 2018 classification.

The mean TLPD of 0.06 demonstrated in this study may seem to be a favorable result when compared with similar studies that have shown a wide range of values from 0.01 to 0.36 teeth/patient/year (mean 0.12)^30^. Considering the fact that even teeth assessed to be hopeless or questionable can be retained for a relatively long time when appropriate SPT is provided^19^, appropriate SPT and periodontitis control by periodontal specialists should play an important role in preserving teeth and preventing TLPD.

Negative binomial regression showed that grade C had the highest incidence rate ratio during the whole treatment period, the ATP period, and the SPT period. Because grade represents disease progression, it is thought that the longer the SPT, the lower is the association of grade at the initial diagnosis with TLPD during SPT. Grade determined at the initial diagnosis should be considered as just one of the predictors at that time. When the treatment period becomes longer, the grade should be re-determined occasionally and the prognosis after the time of re-determination should be re-evaluated to provide a more accurate prognosis of periodontal disease based on the new 2018 classification.

The proportion of patients in each stage and grade in this study was similar to those obtained in studies conducted at the Christian-Albrechts University in Germany^15^ and at the University of Michigan in the United States^16^, which also investigated the distribution of stages and grades on the basis of the new 2018 classification. In these studies, TLPD rates were the highest in patients in stage IV and grade C, and they were also higher in higher stages and grades. When examining the proportion of patients in the respective grades in the same stage, there were few patients in grade A. In the higher stages, the proportion of patients in grade C was higher than that in grade B. This tendency for the proportion of patients in a higher grade to increase in a higher stage was consistent with the study by Ravidà et al.^16^ Given that the results obtained in this retrospective study in Japanese patients were similar to those obtained in the studies conducted in Germany and the United States, we suggest that the new 2018 classification can be applied to patients globally. By conducting similar epidemiological studies across the world and further accumulating study results, the new classification is expected to increase its importance as an indicator that can be used to determine the prognosis of periodontitis.

## Methods

### Patients

Among the patients who had been treated at a private dental clinic in Japan from 26 August 2005 to 20 June 2021, we selected those for whom we were able to determine a stage, grade, and extent of periodontitis based on the new classification by means of initial interview sheets, oral assessments, periodontal examinations, and full-mouth x-ray images, and who also had received SPT from a periodontist certified by the Japanese Society of Periodontology (JSP) for more than a year. Patients who had been treated for less than a year were excluded.

This study was planned in accordance with the principles of the Helsinki Declaration and conducted after approval by the JSP Ethics Committee (approved on 5 February 2021; approval no. JSP2020001). In addition, in accordance with the Ethical Guidelines for Medical and Health Research Involving Human Subjects, we obtained informed consent from the patients in the form of an opt-out on the website (http://www.owl-dc.jp/privacy.html) and notices in the clinic, and anonymized the information used for this study so that no patient could be identified.

### Data collection: medical history and clinical findings

From the initial interview sheets, oral assessment, and medical records, we obtained information regarding the presence or absence of diabetes; HbA1c level; smoking history and the number of cigarettes smoked per day; sex; age; the number of teeth present at baseline; the total treatment period; TLPD rates during the total treatment period and the main cause of TLPD; probing pocket depth at six points per tooth; mobility by Miller’s classification^31^; the presence or absence of class 2 or more furcations by Lindhe and Nyman’s classification^32^; the presence or absence of secondary occlusal trauma, pathological movement, or flared teeth; and the number of teeth with antagonists. Impacted or retained third molars were excluded from the number of teeth present and TLPD. The ratio of bone resorption to the root length around the most severely affected tooth was measured by Schei’s ruler on the basis of full-mouth dental or panoramic x-ray images at the initial diagnosis.

### Staging and grading based on the new classification

On the basis of the clinical data collected, periodontal specialists determined a stage and grade of periodontitis according to the criteria of the new classification^10^. Owing to insufficient data on clinical attachment loss (CAL), CAL was not included in the diagnostic criteria in this study. Instead, the ratio of bone resorption shown in the dental and panoramic x-ray images was used for staging and grading. After staging, periodontitis was classified as localized (< 30% of teeth involved) or generalized (≥ 30% of teeth involved). In determining the extent, Sanz et al. recently proposed using the percentage of teeth at the stage-defining severity level for providing better clinical information^33^. However in this study, we used the original criteria for the extent and the number of teeth affected by periodontitis, because we aimed to investigate TLPD during APT. Teeth expected to have a hopeless prognosis at the initial diagnosis were included in the number of teeth present for staging.

### Periodontal therapy

All periodontal therapies were performed under the supervision of periodontal specialists. First, in accordance with the conventional method of periodontal therapy, we conducted a periodontal tissue examination, made a diagnosis, and developed a treatment protocol, followed by non-surgical periodontal therapies including oral hygiene instruction, scaling, and root planing by dentists or dental hygienists under the supervision of JSP Board Certified Periodontists. After re-evaluation, periodontal surgical procedures were performed by JSP Board Certified Periodontists when deemed necessary after obtaining consent from the patient. Therapy to restore oral function was also provided when needed. Periodontal surgical procedures included hemisection, root separation, tunneling, and periodontal regenerative therapies using Regroth® or Emdogain® gel. After the completion of APT, patients whose periodontal pocket depth in all teeth was < 3 mm with no bleeding on probing, and patients whose periodontal pocket depth was ≥ 4 mm with no bleeding on probing, furcation involvement, or tooth movement without inflammation underwent SPT in accordance with the JSP Clinical Practice Guideline for Periodontal Treatment 2015^34^. Depending on the periodontal condition and the plaque control of the patients themselves, SPT was performed at intervals of 1–6 months, including oral hygiene instruction, professional mechanical tooth cleaning, scaling, antimicrobial administration in the periodontal pockets, and removal of traumatic factors. When a deep pocket still remained, root planing was performed again. If the periodontitis continued to progress, periodontal pocket curettage or periodontal surgical procedures were performed.

### Statistical analysis

Student’s t-test was used to compare the means between two groups of patients for age, and the Mann–Whitney U test was used to compare the medians between two groups for other variables. In three or more groups, the Kruskal–Wallis and Steel–Dwass tests were used to compare the medians for the tooth number and the annual tooth loss per patient. The chi-square test or Fisher’s exact test was used to compare the proportions. The Shapiro–Wilk test was used to assess the normality of the data. Negative binomial regression analysis with the number of instances of TLPD as a dependent variable was performed. The logarithm of the patient follow-up time was used as an offset variable to adjust for different follow-up times. Easy R (EZR version 1.53)^35^ was used for statistical analysis.

## Supplementary Information

Supplementary Information 1.

Supplementary Information 2.

## Data Availability

Correspondence and requests for material should be addressed to Y.S.
